# Severe fever with thrombocytopenia syndrome complicated with aspergillus endocarditis and multiple organ infarctions after glucocorticoid treatment in an immunocompetent man: a case report

**DOI:** 10.1186/s12879-025-10503-7

**Published:** 2025-01-24

**Authors:** Yuxi Zhao, Xiaoxin Wu, Xinyu Wang, Lanjuan Li

**Affiliations:** State Key Laboratory for Diagnosis and Treatment of Infectious Diseases, National Clinical Research Center for Infectious Diseases, National Medical Center for Infectious Diseases, Collaborative Innovation Center for Diagnosis and Treatment of Infectious Diseases, The First Affiliated Hospital, Zhejiang University School of Medicine, 79 Qingchun Rd., Hangzhou City, 310003 China

**Keywords:** Severe fever with thrombocytopenia syndrome, Infective endocarditis, Invasive pulmonary aspergillosis, SFTS, Bandavirus dabieense, Aspergillus

## Abstract

**Background:**

Severe fever with thrombocytopenia syndrome (SFTS) is an emerging infectious disease characterized by leukopenia and thrombocytopenia, and aspergillosis is a common complication in severe cases. Previous studies have reported cases of SFTS complicated with invasive pulmonary aspergillosis (IPA) and central nervous system aspergillosis. Here, we present the first case of an immunocompetent patient with SFTS who progressed to IPA and *Aspergillus* endocarditis after glucocorticoid treatment, and embolism of the vegetations from the left ventricle led to multiple infarctions in the brain, kidney, and spleen.

**Case presentation:**

A 66-year-old male farmer developed altered mental status during SFTS. His consciousness improved during the treatment of glucocorticoids, intravenous immunoglobulin, and ribavirin, but he developed embolisms in the spleen and right kidney, initially attributed to atrial fibrillation, and the anticoagulant agent was not administered due to the high risk of bleeding. Later, He was diagnosed with SFTS-associated IPA (SAPA), for which voriconazole was administered. However, he subsequently experienced a recurrence of altered mental status, accompanied by headache, blindness, and muscle weakness. Brain magnetic resonance imaging (MRI) revealed multiple cerebral embolisms and abscess. The echocardiography showed the vegetations in the left ventricle, suggesting multi-organ embolism caused by infective endocarditis (IE). *Aspergillus fumigatus* was confirmed through pathology and culture of the excised vegetations. The patient was eventually discharged with improved consciousness and muscle strength, but his vision showed minimal recovery.

**Conclusion:**

Clinicians should be wary of aspergillosis in severe patients with SFTS, particularly those receiving glucocorticoid treatment. In patients with SAPA, cerebral aspergillosis and embolic stroke caused by *Aspergillus* endocarditis should also be considered when mental status alters. Furthermore, the possibility of Aspergillosis in other organs should be considered in high-risk patients.

**Supplementary Information:**

The online version contains supplementary material available at 10.1186/s12879-025-10503-7.

## Background

Severe fever with thrombocytopenia syndrome (SFTS) is caused by tick bites carrying *Bandavirus dabieense*, presenting with hyperpyrexia, thrombocytopenia, and leukopenia [1]. SFTS can lead to various complications, often associated with fatal outcomes.

SFTS-associated encephalopathy/encephalitis (SFTSAE) is linked to cytokine storms and the neurotropism of *Bandavirus dabieense*, presenting with a wide spectrum of reversible central nervous system (CNS) symptoms such as lethargy, confusion, dysphoria, suggesting a severe condition and poor prognosis [2]. Electroencephalogram typically shows a slow background rhythm [3]; however, survivors generally do not exhibit evident neurological sequelae [4, 5].

Secondary infections, particularly pulmonary infections, are common in SFTS. The fungi isolated from the respiratory tract are mainly *Aspergillus* species, primarily *Aspergillus fumigatus* and *Aspergillus flavus*, which can lead to aspergillosis [6, 7, 8]. Aspergillosis generally occurs in immunocompromised individuals [9], but it is not a rare complication among patients with SFTS. The incidence of SFTS-associated invasive pulmonary aspergillosis (SAPA) ranges from 10.2 to 31.9%, with mortality rates fluctuating between 26.6% and 53.3% among patients with SFTS [8]. SAPA commonly manifests as airway ulceration covered by pseudomembranes during bronchoscopy, and nodules in chest scans [8, 10]. *Aspergillus* exhibit both vascular and airway invasiveness [11]. Besides SAPA, a case of CNS aspergillosis has also been reported [12]. However, there have been no reported cases of SFTS complicated with *Aspergillus* endocarditis until now. Here, we present a case of an immunocompetent patient with SFTSAE progressed to SAPA and subacute *Aspergillus* endocarditis after glucocorticoid treatment. Ultimately, embolism of the vegetations from the left ventricle led to multiple infarctions in the brain, kidney, and spleen.

## Case presentation

A 66-year-old male farmer with a medical history of paroxysmal atrial fibrillation (AF) and hypertension was admitted to a local hospital due to the fever (highest temperature of 40.9 °C) and diarrhea (5–6 times/day). He was diagnosed with severe fever with thrombocytopenia syndrome by detecting the *Bandavirus dabieense* RNA in the serum through real-time PCR. Six days after symptom onset, the patient developed confusion, lethargy, urinary incontinence, with oral bleeding, and was transferred to our hospital.

**Fig. 1:**
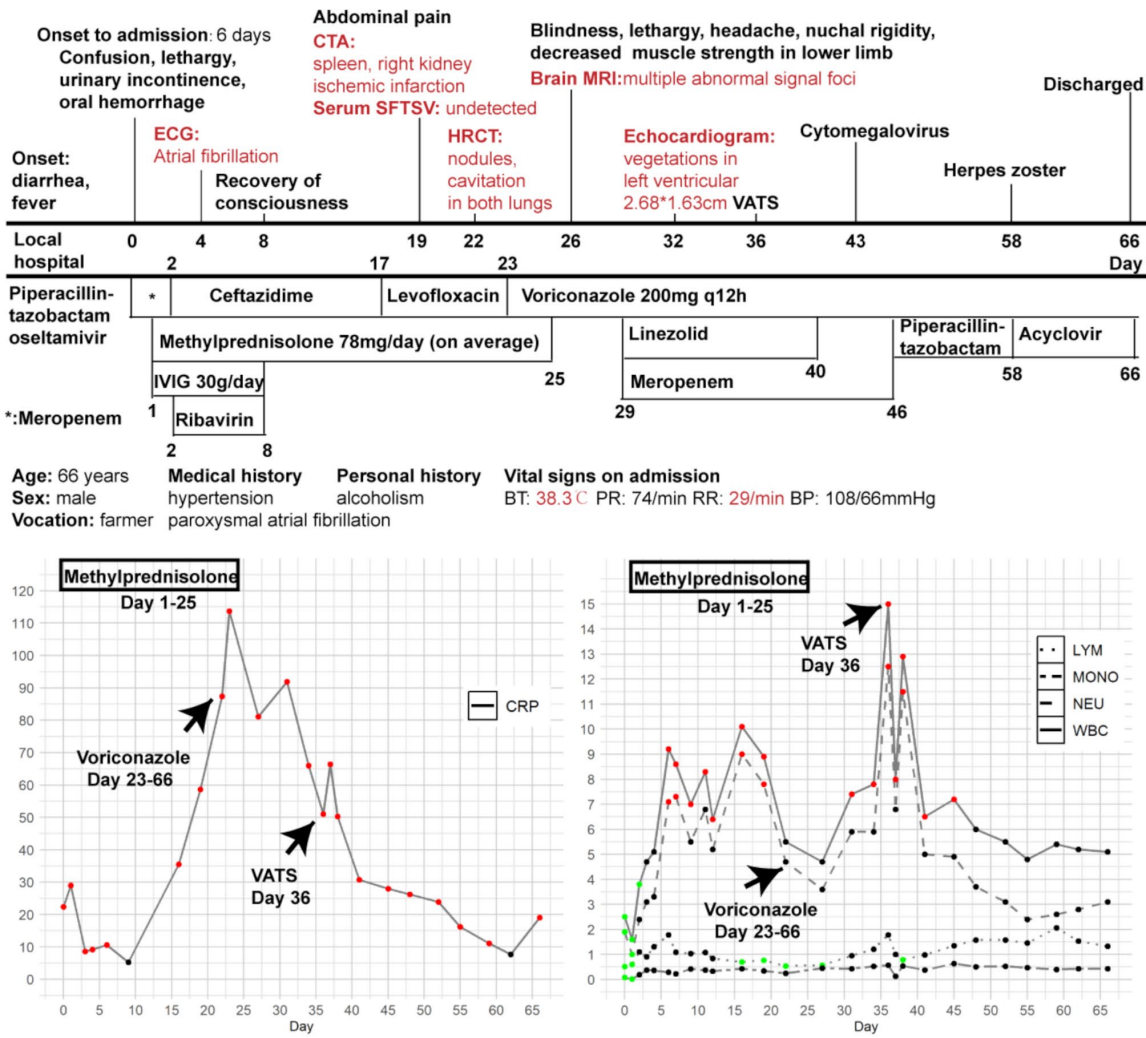
Therapeutic process and changes in CRP levels and complete blood count from admission to discharge **Notes**: The red, green, and black dots represent values > upper limit of normal (ULN), < lower limit of normal (LLN), and normal, respectively **Abbreviations**: BP, blood pressure; BT, body temperature; CRP, C reactive protein; CTA, computed tomography angiography; ECG, electrocardiogram; HRCT, high-resolution computed tomography; IVIG, intravenous immunoglobulin; LYM, lymphocyte; MONO, monocyte; MRI, magnetic resonance imaging; NEU, neutrophil; PR, pulse rate, RR, respiratory rate; SFTSV, severe fever with thrombocytopenia syndrome virus; VATS, video-assisted thoracoscopic surgery; WBC, white blood cell

On admission (Day 0), several laboratory values significantly exceeded the upper limit of normal (ULN): activated partial thromboplastin time: 46 s (ULN: 33.5 s), thrombin time: 26.5 s (ULN: 21.5 s), D-dimer: 31,552 µg/L FEU (ULN: 700 µg/L), cardiac troponin I: 0.162 ng/mL (ULN: 0.060 ng/mL), ferritin: >40,000 ng/mL (ULN: 323 ng/mL), serum creatinine: 151 µmol/L (ULN: 111 µmol/L), indicating the multiple organ dysfunction syndrome. Tests for SARS-CoV-2, malaria, tuberculosis, viral hepatitis, syphilis, human immunodeficiency virus, cytomegalovirus, and Epstein Barr virus were all negative. Serum Immunoglobulin levels were within the normal range. The first cerebrospinal fluid testing (Table 1), electrocardiography, echocardiography, chest imaging and brain magnetic resonance imaging (MRI) showed no significant findings. The therapeutic process, changes in the levels of C-reactive protein (CRP) levels and complete blood count are depicted in Fig. 1.

**Table 1:** Cerebrospinal fluid testing results after two episodes of altered mental status

Variables	First testing	Second testing
	Day 2	Day 26
Opening pressure, mmH_2_O	145	170
Appearance	Clear	Clear
Pandy test	Negative	Positive
ADA, U/L	1	0.5
LDH, U/L	33	79
TNC, /ul	1	8
RBC, /ul	5	2880
Glucose, mmol/L	7.3	3.5
Chlorine, mmol/L	133	115
Protein, g/L	0.38	0.49
Immunoglobulin G, mg/dL	ND	16.7
Albumin, mg/dL	ND	21.7

**Abbreviations**: ADA, adenosine deaminase; LDH, lactate dehydrogenase; ND, not done; RBC, red blood cell; TNC, total nucleated cells

**Fig. 2:**
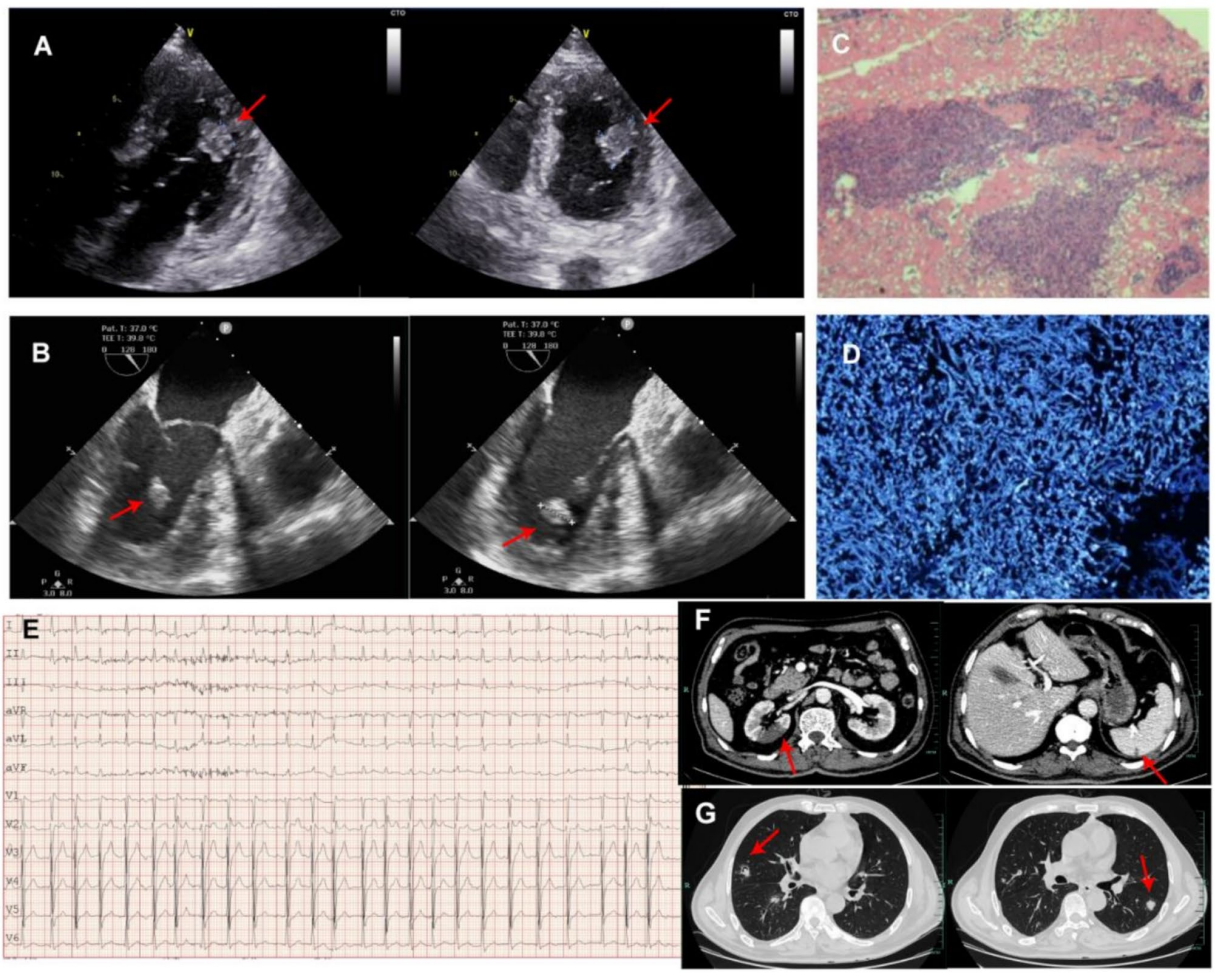
(**A**) Echocardiography: The vegetation (2.68 × 1.63 cm) was detected at the junction of the posterior-lateral edge of the anterior papillary muscle and the left ventricular lateral wall, the base (1.13 cm) was attached to the endocardium of the lateral wall. The lobulated distal portion moved with the cardiac cycle. (**B**) Transesophageal echocardiography: Several hypoechoic vegetations were detected on the anterior papillary muscle of the left ventricle, with the largest measuring 1.6 × 1.4 × 1.1 cm. The base was fixed to the papillary muscle, and it moved with the cardiac cycle. (**C**) Pathology of vegetations by hematoxylin and eosin stain. (**D**) Identification of Aspergillus by fluorescent in situ hybridization. (**E**) Electrocardiogram: atrial fibrillation with rapid ventricular rate. (**F**) Abdominal computed tomography angiography: right kidney with ischemic infarction and spleen with subcapsular infarction. (**G**) High-resolution computed tomography: Crescent signs and nodules in the lungs

The patient was diagnosed SFTSAE and treated with intravenous immunoglobulin (IVIG), methylprednisolone, and ribavirin. Digitalis and metoprolol were administered due to the AF with a rapid ventricular rate (Day 4) (Fig. 2), but were withdrawn due to the bradycardia (Day 6). On Day 8, his mental status improved, and he could respond with simple words.

On Day 19, the patient developed persistent mild abdominal pain. Abdominal computed tomography angiography revealed infarctions in the right kidney and spleen (Fig. 2). The embolism was presumed to be cardiogenic caused by AF. However, due to thrombocytopenia and coagulation dysfunction, the risk of hemorrhagic events was high, and therefore, anticoagulant agent was not administered.

Although *Bandavirus dabieense* was undetected in the serum, CRP levels continued to rise. Therefore, the chest high-resolution computed tomography scan was performed on Day 22, and showed crescent sign and multiple nodules in both lungs (Fig. 2). Subsequently, the serum (1,3)-beta-D-glucan test (G-test) and galactomannan (GM-test) were conducted, with results of 494.09 pg/mL (ULN: 60.00 pg/mL) and 0.25 µg/mL (ULN: 0.50 µg/mL), respectively. Given the clinical diagnosis of SAPA, voriconazole 200 mg every 12 h was started (Day 23) and continued until discharge.

**Fig. 3:**
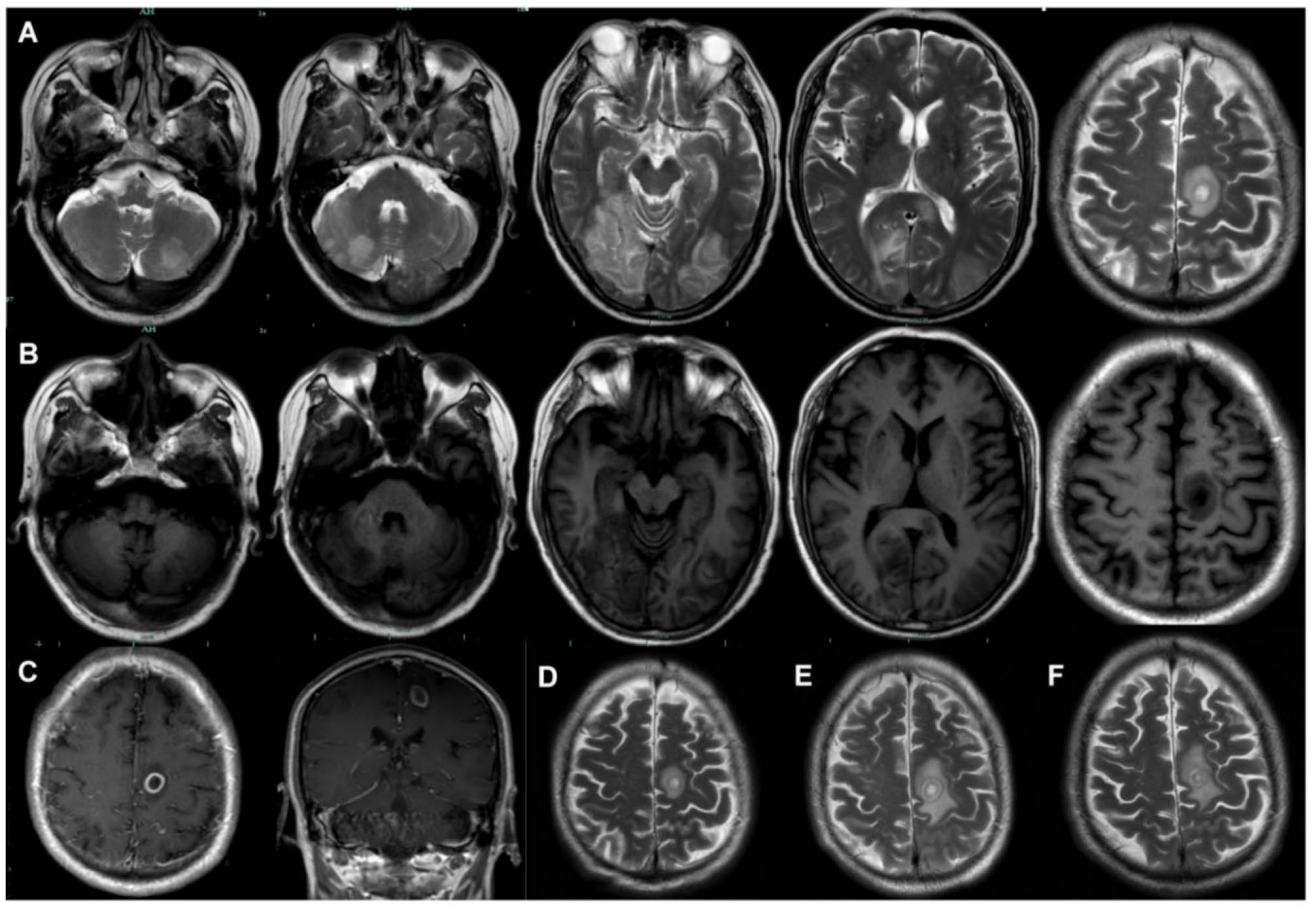
Multiple signal abnormalities in the bilateral cerebellum, frontal, temporal, parietal, occipital lobes, and corpus callosum. (**A**) T2-weighted imaging. (**B**) T1-weighted imaging. (**C**) T1-weighted contrast-enhanced imaging: the abscess with a diameter of 13.1 mm, surrounded by a hyperintense ring with peripheral patchy hypointensity. (**D-F**) Axial T2-propeller imaging: the abscess following stroke on day 0, day 21, day 35

On Day 26, the patient experienced a sudden loss of vision in both eyes (light perception only), accompanied by lethargy, severe headache upon positional change, and bilateral lower limb muscle weakness (Grade 4). Nuchal rigidity was also observed. The lumbar puncture was conducted again (Table 1). Brain MRI showed abscess and multiple signal abnormalities (Fig. 3). CNS infection and cortical blindness were considered. On Day 29, the G-test was 211.71 pg/mL, and the GM-test was 1.64 µg/mL, but *Bandavirus dabieense* remained undetected in the serum.

Despite the treatment of meropenem, linezolid and voriconazole, the G test was 212.46 pg/mL (Day 31), and the patient continued to experience daily febrile episodes (up to 38.7 °C), high levels of CRP, but repeated blood cultures for bacteria and fungi were negative. Combined with the history of multi-organ embolism, infective endocarditis (IE) was suspected. The echocardiogram was conducted and showed left atrial enlargement (5 × 6 cm), left ventricular hypertrophy (Wall thickness: 1.6 cm), and the vegetations in the left ventricle (Fig. 2) (Supplementary material 1).

On day 36, the patient underwent video-assisted thoracoscopic surgery. During the surgery, a large amount of pale yellow pericardial fluid was observed. A large vegetation (3 cm) and two smaller vegetation (1 cm) were removed from the left ventricle. None of the vegetations were attached to the mitral valve. The intraoperative frozen section revealed inflammatory necrotic tissue with fungal clusters, and the fungal morphology was consistent with *Aspergillus* species. Cultures of the vegetations subsequently identified *Aspergillus fumigatus*.

Although cytomegalovirus DNA was detected in the serum (Day 43), the fundus examination revealed only cotton-wool spots, thereby excluding the cytomegalovirus retinitis. The patient’s mental status and muscle strength gradually improved after surgery, but his vision showed minimal recovery. He was eventually discharged (Day 66).

The serum voriconazole trough concentrations on days 30, 32, 50, and 64 were 6.5, 4.0, 2.3, and 1.5 µg/mL, respectively.

## Discussion

To our knowledge, this is the first case report of SAPA complicated by subacute *Aspergillus* endocarditis. The patient experienced two episodes of alerted mental status. The first episode was associated with SFTSAE, while the second was primarily attributed to the stroke and intracranial fungal infection caused by embolization from fungal vegetations in the left ventricle.

Cases with SFTSAE reported often treated with high-dose glucocorticoids and IVIG [13, 14], however, previous reports suggest that glucocorticoid treatment does not improve SFTS prognosis and increases the risk of secondary infections, including SAPA [8, 15, 16]. IVIG has also shown limited benefits and may even cause immune disorders [17]. Additionally, in the early stages of SFTS, leukopenia, and impairment in both innate and adaptive immunity caused by virus and cytokine storms were observed [18, 19], and as such, patients are at high risk of aspergillosis [6, 7, 20]. Novel immunomodulatory drugs may be the effective alternatives in improving prognosis and reducing secondary infections among severe cases of SFTS. A single-arm trial demonstrated improvements in neurological symptoms, laboratory parameters, and 28-day overall survival in patients treated with ruxolitinib compared to the historical control group [21]. Furthermore, in a randomized clinical trial, tocilizumab significantly reduced the mortality rate, without significant differences in the incidence of secondary infections [22].

Both invasive pulmonary aspergillosis (IPA) and CNS aspergillosis occurred in this case. For IPA in non-neutropenic patients, high-resolution CT and bronchoalveolar lavage GM-test are the primary diagnostic methods, and for CNS aspergillosis, brain MRI and CSF GM-test are valuable tools [9]. The CSF GM-test has a positive predictive value ranging from 69 to 93.8% and a sensitivity of 80% [23, 24, 25, 26], and the positivity rate of serum GM-test is higher in patients with hematogenous dissemination compared to those with contiguous dissemination [26]. Voriconazole is the primary therapy for both IPA and CNS aspergillosis, and therapeutic drug monitoring is recommended, with serum trough concentrations 2–6 mg/L for CNS infections [9, 27].

However, this case was also complicated with *Aspergillus* endocarditis. According to the updated modified Duke criteria [28], definite endocarditis was diagnosed in this case based on both pathologic criteria (*Aspergillus* in vegetations) and clinical criteria. The clinical criteria were met with two major criteria: imaging major criterion (vegetation seen on echocardiogram) and surgical major criterion (direct inspection), and two minor criteria: fever (> 38 °C) and vascular phenomena (arterial emboli and cerebral abscess). Fungal endocarditis accounts for 1–3% in all IE cases, and *Aspergillus* species accounts for approximately a quarter in fungal endocarditis cases, risk factors include prosthetic valves, heart surgery history, injection drug use and corticosteroids use [29, 30, 31, 32]. *Aspergillus* endocarditis is a significant predictor of the fatal outcome in fungal endocarditis, with blood cultures showing a 28.0% positivity rate, much lower than Candida’s 88.9% [32]. The diagnostic value of serum GM-test for fungal endocarditis remains inconclusive [32], and it is not specific for the site of disease [9]. Transesophageal echocardiography is the primary imaging technique [31], and early surgical intervention combined with antifungal therapy is the major therapeutic strategy [9].

AF is common in SFTS patients [33]. According to the CHA2DS2-VASc score [34], this case met two criteria—age (65–74) and hypertension, and the total points was 2, indicating the high risk for thromboembolism. Initially, we suspected that the first embolic event (EE) might be attributed to AF. However, upon further review, we noted despite negative serum *Bandavirus dabieense* result and the use of antibiotics, CRP levels continued to rise rapidly after the first EE, suggesting a fungal infection. The occurrence of second EE and brain abscess strongly indicated IE. While both AF and IE can lead to arterial emboli, IE is also associated with fever, infection at the embolic sites, sepsis, immunologic phenomena, the presence of a new murmur and valvular decompensation [9, 28]. Additionally, risk factors for IE-related embolism may include vegetation size > 10 mm, vegetation mobility, and CRP levels > 40 mg/L in this case [35].

The absence of follow-up imaging limited our ability to dynamically assess the treatment response. In aspergillosis, monitoring lesion changes through follow-up imaging is crucial for evaluating treatment efficacy, identifying possible complications and recurrence. It is also an important basis for guiding medication regimens and further intervention including surgery.

## Conclusion

Clinicians should be wary of aspergillosis in severe patients with SFTS, particularly those receiving glucocorticoid treatment. In patients with SAPA, when mental status alters, in addition to considering SFTSAE, CNS aspergillosis and embolic stroke caused by *Aspergillus* endocarditis should also be considered, and echocardiography is significant in differential diagnosis. Furthermore, the possibility of Aspergillosis in other organs should be considered in high-risk patients.

## Electronic supplementary material

Below is the link to the electronic supplementary material.

Supplementary Material 1

## Data Availability

All data supporting the findings of this study are available within the paper and its Supplementary Information.

## Abbreviations

CNS: Central nervous system
CRP: C-reactive protein
EE: Embolic event
G-test: (1,3)-beta-D-glucan test
GM-test: Galactomannan test
IE: Infective endocarditis
IPA: Invasive pulmonary aspergillosis
IVIG: Intravenous immunoglobulin
MRI: Magnetic resonance imaging
SAPA: SFTS-associated IPA
SFTS: Severe fever with thrombocytopenia syndrome
SFTSAE: SFTS-associated encephalopathy/encephalitis
ULN: Upper limit of normal
